# Contraception in the Era of COVID-19

**DOI:** 10.9745/GHSP-D-20-00119

**Published:** 2020-06-30

**Authors:** Kavita Nanda, Elena Lebetkin, Markus J. Steiner, Irina Yacobson, Laneta J. Dorflinger

**Affiliations:** a FHI 360, Durham, NC, USA.

## Abstract

As global health systems and communities prepare to meet an unprecedented threat causing increased demands for the care of people with COVID-19, health care providers should strive to ensure continuity of reproductive health care to women and girls in the face of facility service disruption.

## Key Messages

- In the face of facility service disruptions due to COVID-19, health care providers, particularly in low- and middle-income countries, should strive to maintain continuity of reproductive health care to women and girls as an essential service.
- When in-person encounters are limited, health care providers should adapt the way contraceptive services are provided by using telehealth whenever possible for counseling, shared decision making, and side effect management, and should make adjustments to provision of contraceptive methods to ensure access.

Globally, approximately 50% of pregnancies are unintended.^1^ In low- and middle-income countries, where access to health care may be limited, unintended pregnancies can have dire consequences ranging from unsafe abortion to serious pregnancy complications that contribute to maternal and infant mortality.^2^ As such, contraception is lifesaving and an essential component of reproductive health care. The ability to access and continue using contraception improves women’s reproductive autonomy, reduces unintended pregnancies, and profoundly impacts both women’s and family’s lives, health, empowerment, and well-being, particularly in times of stress and hardship.

As medical systems, clinics, and communities prepare to meet an unprecedented threat causing increased demands for the care of people with COVID-19, strategies to mitigate virus spread and optimize health care resources are evolving and will need to be country specific. Health care providers should strive to ensure continuity of reproductive health care to women and girls in the face of facility service interruption. Even while annual exams and nonurgent appointments are canceled, maintaining access to reproductive health services, including provision of contraception, is key to a comprehensive COVID-19 mitigation strategy and to sustaining the successes of high-quality family planning services that contribute to lowering maternal mortality and improving newborn and child health.

The World Health Organization (WHO) recognizes that countries are at different stages of the COVID-19 epidemic/transmission scenario: (1) no reported cases, (2) sporadic cases, (3) clusters of cases, or (4) community transmission. However, public health measures that WHO recommends for all 4 scenarios include social distancing.^3^ To ensure continuation of contraceptive access and services, including counseling and shared decision making, a number of adaptations to existing systems are required. In particular, maximizing the use of a “no-touch” approach to care whenever possible is essential.

## USE TELEHEALTH FOR COUNSELING AND SCREENING

Use various communication methods that do not require in-person contact (SMS, WhatsApp, video calls, or telephone calls) to:

- Counsel new clients requesting contraception and to screen for medical eligibility.^4^^–^^6^
- Issue new prescriptions and refills for clients who desire user-controlled contraceptives (e.g., combined oral contraceptives, progestin-only pills, contraceptive patches, or vaginal contraceptive rings) if no contraindications are evident. Send all prescriptions directly to the pharmacy or clinic to limit contacts.
- Inform clients who desire long-acting reversible contraceptives (LARCs) of service locations where LARCs are being provided.
- Manage and treat contraceptive side effects, if possible.

## PROVIDE ADDITIONAL COUNSELING AND INFORMATION

- Counsel on fertility awareness methods^7^ and correct and consistent condom use in case disruptions occur in the supply of other contraceptive commodities.
- Counsel current LARC users on the effectiveness of extended use beyond the labeled duration, postponing routine removals.^8^
- Educate clients on emergency contraception including both over-the-counter and prescription options.

## OPTIMIZE HOW CLIENTS ACCESS CONTRACEPTIVE METHODS

- Prescribe/dispense multimonth refills to minimize trips to the pharmacy or clinic. Health insurance plans (where existing) should waive time limitations on refills to allow for multimonth dispensing and should consider eliminating or decreasing prescription costs.
- Train for and offer self-injection of Sayana Press (DMPA-SC), where available, for women desiring injectable contraception.^9^
- Continue to offer insertion of LARC methods, such as intrauterine devices and contraceptive implants, to new users where possible with adequate safety preparations for the procedure. Make arrangements to avoid having too many clients in the waiting area. This may involve scheduling clients individually, having clients wait outside, and/or ensuring clients maintain adequate social distancing precautions while inside. If LARC insertion is unavailable, offer the client user-controlled methods.
- Limit direct contact with current LARC users to situations where removal cannot be delayed or when side effects require a physical/pelvic exam or other tests.
- Consider placing clients who desire permanent contraception on waitlists and offering them bridge contraception as operating rooms ramp down all but the most urgent surgery.
- Provide advance prescriptions for emergency contraception to increase awareness and reduce barriers to immediate access.

## MAKE CONSIDERATIONS FOR POSTPARTUM WOMEN

Where possible, initiate or continue counseling and access to immediate postpartum contraception before hospital discharge, particularly as access to postpartum visits becomes limited.

- Provide LARC immediately postpartum for clients who desire LARC and are eligible.^6^^,^^10^
- Perform permanent contraception procedures for clients who desire it at the time of cesarean delivery and/or after vaginal delivery, if available.
- Counsel on correct use of the lactational amenorrhea method.
- Administer DMPA, if the client desires.
- Prescribe or dispense user-controlled contraceptive methods, including Sayana Press where available, in sufficient quantities to be initiated or continued at home by women who are not breastfeeding or, if breastfeeding, initiated as soon as one of the lactational amenorrhea method criteria expires. Provide method-specific instructions to delay until the client is medically eligible per WHO criteria.

As the world grapples with the novel COVID-19 pandemic, we in the public health community must continue to provide guidance and support to ensure that all women, men, and adolescents can access safe and affordable contraception and contraceptive services. Adjustments to the way services are provided are inevitable; however, quality of and access to services must be maintained. We believe the above guidance provides a foundation for continuing safe contraceptive service provision that countries may adapt to the local context, taking into consideration local policies and stage of the epidemic in each country.

This statement has been adapted from the American College of Obstetricians and Gynecologists (ACOG) COVID-19 FAQs for Obstetrician-Gynecologists, Gynecology, available at https://www.acog.org/clinical-information/physician-faqs/covid19-faqs-for-ob-gyns-gynecology.

## Notes

**Peer Reviewed**

**First published online:** April 20, 2020
